# Nervous system and ciliary structures of Micrognathozoa (Gnathifera): evolutionary insight from an early branch in Spiralia

**DOI:** 10.1098/rsos.160289

**Published:** 2016-10-26

**Authors:** Nicolas Bekkouche, Katrine Worsaae

**Affiliations:** Marine Biological Section, Department of Biology, University of Copenhagen, Universitetsparken 4, 2100 Copenhagen, Denmark

**Keywords:** *Limnognathia maerski*, meiofauna, neuro-morphology, retrocerebral organ, acetylated α-tubulin, serotonin

## Abstract

Recent studies show that Gnathifera, comprising Rotifera, Gnathostomulida and Micrognathozoa, constitute the sister group to the remaining Spiralia (containing, e.g. flatworms, segmented worms and molluscs). Therefore, a better understanding of Gnathifera is central for unravelling the evolution of the highly diverse Spiralia. Here, we describe the previously unstudied nervous system and ciliary structures of Micrognathozoa, using immunohistochemistry and confocal laser scanning microscopy. The nervous system is simple with a large brain, paired sub-esophageal ganglia, two trunk commissures, two pairs of ventral longitudinal nerves and peripheral nerves. The paired ventro-lateral nerve cords are confirmed to be a symplesiomorphy of Gnathifera (possibly even Spiralia), whereas the paired ventro-median nerves are not previously reported in Gnathifera. A pharyngeal ganglion is described for Micrognathozoa: a complex structure with two apical tufts of ciliary receptors, now shown to be shared by all Gnathifera. The ventral pattern of external ciliophores is re-described, and protonephridia with multi-ciliated collecting tubules similar to those of Rotifera are confirmed. A range of new details from a simple nervous system and complex set of ciliary structures in a microscopic metazoan are hereby unravelled. The many resemblances with Rotifera corroborate their close relationship, and shed more light on the evolution of Gnathifera.

## 1. Introduction

*Limnognathia maerski* (Kristensen and Funch, 2000) (Micrognathozoa) [1] is a recently described species, belonging to the bilaterian clade ‘Gnathifera’. Recent phylogenomic studies show that Gnathifera is probably the sister group of remaining spiralians, and therefore is of crucial importance to understand animal evolution [2,3]. However, studies on the different organ systems of Gnathifera are still warranted. Indeed, this clade is constituted of small, sometimes rare animals, the collection of which is difficult and time-consuming, namely Gnathostomulida, Rotifera (= Syndermata, including Acanthocephala) and Micrognathozoa. The deep interrelationships between these three lineages is now resolved with both phylogenomics [3] and morphology [1,4,5], supporting a sister group relationship between Micrognathozoa and Rotifera, and Gnathostomulida being sister group to this clade. Although rotifers are relatively well studied in many aspects, most of their internal morphology still needs further investigation, as it is the case for the internal organization in Gnathostomulida and Micrognathozoa.

Several confocal laser scanning microscopy (CLSM) studies have been conducted on gnathiferans, but most of them have focused on the musculature, e.g. in rotifers [6–8], one genus of Gnathostomulida [9,10] and Micrognathozoa [11]. On the other hand, CLSM studies on the nervous system of Gnathifera are quite scarce (e.g. for rotifers [12–15] and for gnathostomulids, [10,16]), and no studies have yet been carried out on Micrognathozoa. According to previous studies, the nervous system of Gnathostomulida consists of an anterior brain, a buccal ganglion, an anterior and a posterior commissure, and a variable number of longitudinal nerves extending along parts of the entire body length (three paired and two unpaired in *Gnathostomula paradoxa* Ax, 1956 [10,17], six pairs in *Rastrognathia macrostoma* Kristensen & Nørrevang, 1977 [18], and three paired and two unpaired in *Pterognathia meixneri* Sterrer, 1969 [19,20]). Most studied rotifers show the presence of a brain, a mastax ganglion, a pair of ventro-lateral nerve cords as well as various head and peripheral nerves innervating the muscles and the sensory organs. However, extensive studies of the nervous system of rotifers are rare, and most recent publications focused on specific immunoreactivity (IR) [12–14].

Micrognathozoa were first collected from a cold freshwater spring in 1994 in Greenland [1], and thereafter reported from sub-antarctic islands [21], and the United Kingdom [11]. But specimens from the United Kingdom are extremely rare, and the sub-antarctic islands as well as Greenland are remote localities, making the study of fresh material difficult. These ventrally ciliated meiofaunal animals, measuring up to 150 µm in length comprising a head, thorax and abdomen, have very complex jaws, and only females are known so far. The complexity of the jaws (sclerites) has attracted most of the attention, and together with the original description, the sclerite arrangement has been described in detail [1,21,22], but recently, the musculature of Micrognathozoa was finally resolved [11].

The nervous system was superficially addressed in the original description [1], and therein described as comprising a bilobed brain connected to a pair of ventro-lateral nerve cords with two paired ganglia (in the thorax and in the posterior-most part of the abdomen). Furthermore, the presence of a buccal ganglion is suspected, but not confirmed [23]. The ventral ciliation was described as consisting of a dense head ciliation, four pairs of head ciliophores, 18 pairs of ciliophores arranged in two mid-ventral longitudinal rows and a posterior adhesive ciliary pad. Moreover, two pairs of protonephridia were originally described in the thorax [1] with later discussion on the possible opening of their canal cells into a common collecting tubule [23] and the location of the nephridiopore remaining unknown.

The nervous system and ciliary patterns of *L. maerski* (Micrognathozoa) are here described in detail, using CLSM and immunohistochemistry, in order to understand the structure and evolution of these different organ systems within Gnathifera, the sister group to the remaining Spiralia.

## 2. Material and methods

### 2.1. Collection of specimens

Mosses were collected at the type locality in the Isunngua Spring on Disko Island, West Greenland, 69°43′ N 51°56′ W, on 31 July 2013. The mosses were squeezed into a 32 µm mesh, and the extract was thereafter sorted using dissecting scopes, picking up the animals alive with a pipette or an Irwin loop.

### 2.2. Immunohistochemistry and confocal laser scanning microscopy

Specimens were anaesthetized with 1% magnesium chloride solution added drop by drop until no movements were visible and fixed in 3.7% paraformaldehyde in phosphate buffered saline (PBS) for 1–2 h at room temperature (RT), followed by six rinses in PBS and storage in PBS with 0.05% NaN_3_. For the investigation of the muscular, nervous, glandular and ciliary system, triple or quadruple staining was applied, including F-actin staining (Alexa Fluor 488-labelled phalloidin, INVITROGEN, Carlsbad, USA), DNA-staining (405 nm fluorescent DAPI) and antibodies against neurotransmitters and tubulin (monoclonal mouse anti-acetylated α-tubulin (SIGMA T6793, St Louis, USA), polyclonal rabbit anti-serotonin (5-HT, SIGMA S5545) and anti-FMRF-amide (IMMUNOSTAR 20091, Hudson, USA)). Prior to adding the primary antibody-mix, the samples were preincubated with 1% PTA (PBS + 0.1% Triton-X, 0.05% NaN3, 0.25% BSA, and 5% sucrose) for 1 h. Samples were incubated over night at RT in primary antibodies (mixed 1 : 1 with glycerol) in a final 1 : 400 concentration. Subsequently, specimens were rinsed in PBS six times and incubated with the secondary antibodies conjugated with fluorochromes overnight at RT (mixed 1 : 1 with glycerol) in a final concentration of 1 : 400; goat anti-mouse conjugated with CY5 (JACKSON IMMUNO-RESEARCH, West Grove, USA, 115-175-062), goat anti-mouse conjugated with FITC (JACKSON IMMUNO-RESEARCH, West Grove, USA, 115-175-062) and goat anti-rabbit labelled with TRITC (SIGMA T5268). Afterwards, they were rinsed in PBS five times and preincubated for 60 min in Alexa Fluor 488-labelled phalloidin (0.33 M in 1% PTA). Thereafter, specimens were rinsed in PBS (without NaN_3_) and mounted in Fluoromount-G with DAPI (Southern Biotechnology Associates, Inc., Alabama, USA) or Vectashield with DAPI (Vector Laboratories, Burlingame, USA). The specificity of the antibodies was tested by omitting each of the primary and secondary antibodies.

The mounted specimens were scanned using an Olympus Fluoview FV-1000 confocal laser scanning microscope (of K. Worsaae, University of Copenhagen, Denmark) or a Leica TCS SP5 CLSM, with the acquired z-stacks of scans being either projected into two-dimensional images or analysed three-dimensionally using IMARIS v. 7.1 (Bitplane Scientific Software, Zürich, Switzerland). This software package was also used to conduct the measurements presented in the following text. Schematic drawings and plate set-up were done with Adobe Illustrator CS6, and image adjustments were conducted in Adobe Photoshop CS6.

## 3. Results

### 3.1. Nervous system

**Figure 1.**
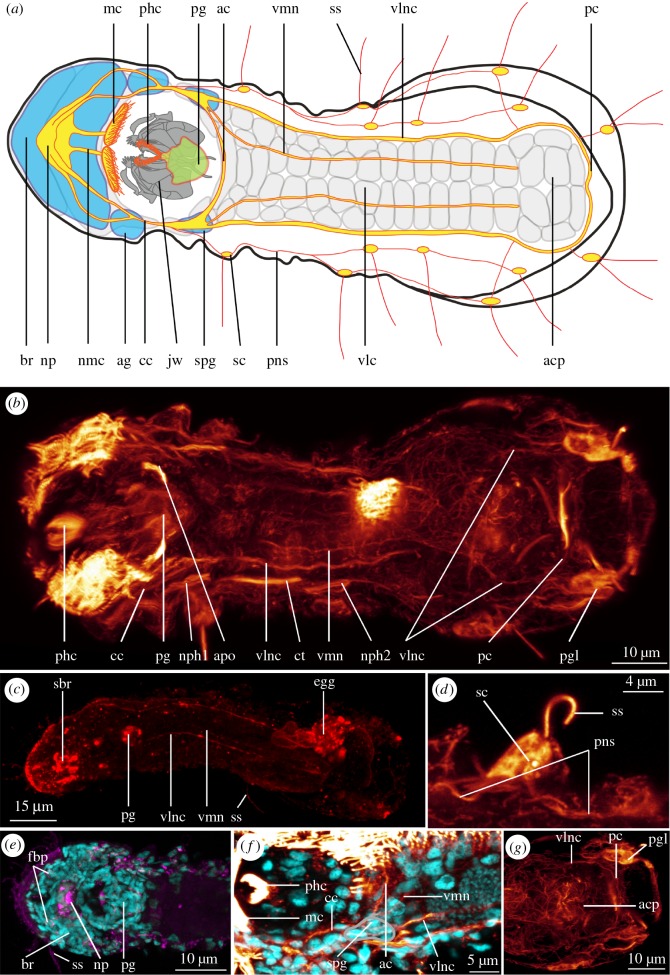
Nervous system of *Limnognathia maerski*. (*a*) Schematic drawing of the nervous system of *L. maerski*. Structures recognized with DAPI in blue, acetylated α-tubulin-LI-reactive nervous system in orange/yellow, and locomotory ciliation in light grey. (*b*–*g*) CLSM maximum intensity projection. Acetylated α-tubulin-LIR in glow, serotonin-LIR in red, FMRF-amide-LIR in purple and DAPI in cyan. (*b*) General overview of the nervous system. Note that some deformation occurred during scanning, resulting in an artefactual elongation of the pharyngeal ganglion. (*c*) General overview of the serotonin-LI-reactive nervous system. (*d*) Details of sensoria and peripheral nervous system. (*e*) Overview of the FMRF-amide-LI-reactive brain and pharyngeal ganglion. (*f*) Details of the anterior commissure and sub-pharyngeal ganglion. (*g*) Details of the posterior commissure. Anterior end of specimens pointing left on all figures. ac, anterior commissure; acp, adhesive ciliary pad; ag, auxiliary ganglion; apo, acetylated α-tubulin-LI-reactive pharyngeal organ; br, brain; cc, circumesophageal connective; ct, collecting tubule; egg, egg; fbp, FMRF-amide-LI-reactive brain perikarya; jw, jaw; mc, mouth ciliation; nmc, nerve of the mouth ciliation; np, neuropil; nph1--2, protonephridia 1 and 2; pc, posterior commissure; pg, pharyngeal ganglion; pgl, posterior gland; phc, pharyngeal cilia; pns, peripheral nervous system; sbr, serotonin-LI-reactive brain; sc, sensorium cell body; spg, sub-pharyngeal ganglion; ss, sensorium; vlc, ventral locomotory ciliophores; vlnc, ventro-lateral nerve cord; vmn, ventro-median nerve.

**Figure 2.**
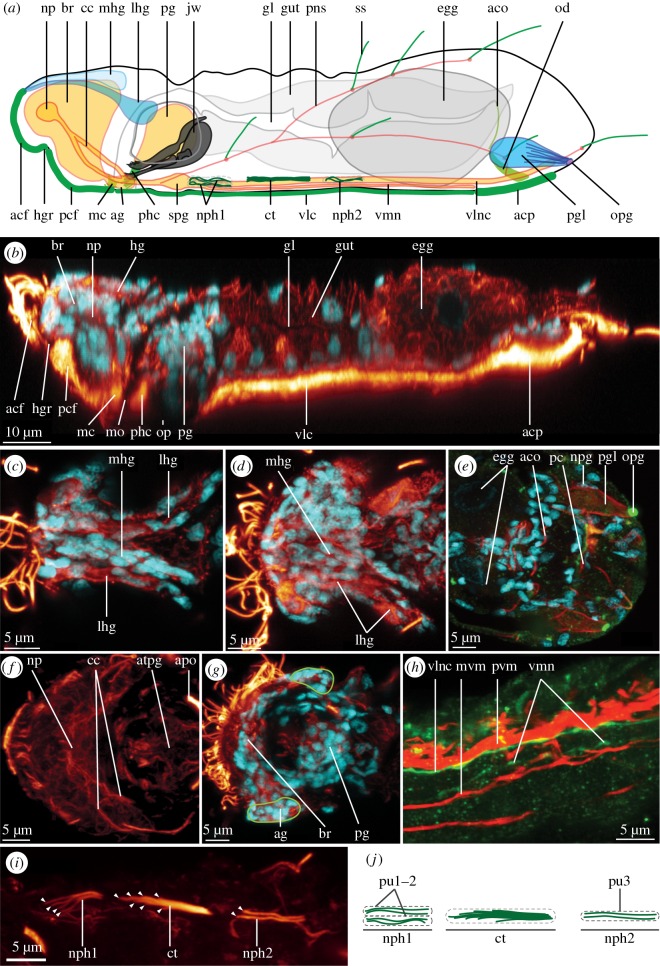
Profile and details of the nervous system and protonephridia in *Limnognathia maerski*. (*a*) Schematic drawing of a lateral view of *L. maerski*. Glandular system in blue, nervous system in orange/yellow and ciliation in green. (*b*–*i*) CLSM maximum intensity projections. Acetylated α-tubulin-LIR in glow, DAPI in cyan, serotonin-LIR in green, and phalloidin in red. (*b*) Virtual mid-sagittal section. (*c*–*d*) Maximum intensity projection of sub-stacks. (*c*) Details of the anterior of the glands of the head. (*d*) Details of the posterior of the glands of the head. (*e*) Details of the posterior glands. (*f*) Details of the acetylated α-tubulin-LI-reactive brain. (*g*) Details of the auxiliary ganglion. (*h*) Details of the relative position of the longitudinal nerves and musculature. (*i*) Close-up of the ciliation of the protonephridial system with arrowheads pointing to the individual cilia. (*j*) Schematic drawing showing the cilia of the terminal cell and the collecting tubule of the protonephridial system. Anterior end of specimens pointing left on all figures. acf, anterior ciliated field; aco, accessory cilia of the oviduct; acp, adhesive ciliary pad; ag, auxiliary ganglion; apo, acetylated α-tubulin-LI-reactive pharyngeal organ; atpg, acetylated α-tubulin-LI-reactive pharyngeal ganglion; br, brain; cc, circumesophageal connective; ct, collecting tubule; egg, egg; gl, gut lumen; gut, gut; hg, head gland; hgr, head groove; jw, jaw; lhg, lateral head gland; mc, mouth ciliation; mhg, median head gland; mo, mouth opening; mvm, median ventral muscle; np, neuropil; npg, nuclei of the posterior gland; nph1,2, protonephridia 1 and 2; od, oviduct; op, oral plate; opg, opening of the posterior gland; pc, posterior commissure; pcf, posterior ciliated field; pg, pharyngeal ganglion; pgl, posterior gland; phc, pharyngeal cilia; pns, peripheral nervous system; pu1--3, protonephridial units 1 to 3; pvm, paramedian ventral muscle; spg, sub-pharyngeal ganglion; ss, sensorium; vlc, ventral locomotory ciliophores; vlnc, ventro-lateral nerve cord; vmn, ventro-median nerve.

The nervous system consists of a large brain, occupying most of the forehead with a dorsal neuropil, two pairs of major longitudinal nerves connected by paired sub-pharyngeal ganglia, an anterior and a posterior commissure, a peripheral nervous system related to the sensory cilia (sensoria), as well as a pharyngeal ganglion (figures 1 and 2).

The nervous system has been investigated with antibodies directed against acetylated α-tubulin, serotonin and FMRF-amide. The quality and strength of the signal of the IR varied substantially between the different specimens examined, even among freshly collected material, with simultaneously fixed and stained specimens. Moreover, in some specimens, for acetylated α-tubulin-like immunoreactivity (LIR) and serotonin-LIR, the signal of the ciliation masks the longitudinal nerves. However, although the acetylated α-tubulin-like-immunoreactive (LI-reactive) signal revealed more or less details in different specimens, it always supports the same pattern. Not all specimens showed clear serotonin-LIR in the nerves, ganglia and brain. In most specimens, FMRF-amide-LIR only shows a clear pattern in the pharyngeal ganglion, and the rest of the signal appears to be unspecific.

#### 3.1.1. Longitudinal nerves

Two pairs of nerves originate from each ventro-lateral side of the brain neuropil, and the two nerves of each side fuse lateral to the pharynx to form the paired circumesophageal connective (cc, figures 1*a*,*f* and 2*a*,*f*), extending ventro-posteriorly to the sub-pharyngeal ganglia (spg, figures 1*a*,*f* and 2*a* described below). The ventro-lateral nerve cords (vlnc, figures 1*a*–*c*,*f*,*h* and 2*a*,*h*) originate from the sub-pharyngeal ganglia, extending throughout the trunk until they connect in the terminal commissure in the posterior abdomen (pc, figures 1*a*,*b*,*g* and 2*e*). Posterior to the pharynx, the nerves are interconnected by the anterior commissure (ac, figure 1*a*,f) of the paired sub-pharyngeal ganglia, the ganglia also supplying the ventro-lateral nerve cords, the circumesophageal connective, and the ventro-median nerve (vmn, figures 1*a*–*c*,*f* and 2*a*,*h*). The presence of one to two pairs of perikarya supplying the ventro-lateral nerve cords is suspected, but could not be confirmed with certainty. The ventro-lateral nerve cords are 1.5 µm thick, and extend along most of the body length, surrounding the adhesive ciliary pad area until the posterior commissure at the posterior margin of the adhesive ciliary pad, where no associated ganglia (clusters of perikarya) could be detected with neither DAPI staining nor the applied neurotransmitters. Co-localization with phalloidin staining shows that the ventro-lateral nerve cords lie adjacent to the paired paramedian ventral muscles (pvm and vlnc, figure 2*h*, and see [11]) and to the lateral margin of the trunk locomotory ciliation. Thus, it is likely that the ventro-lateral nerve cords innervate either one or both of these systems.

**Figure 3.**
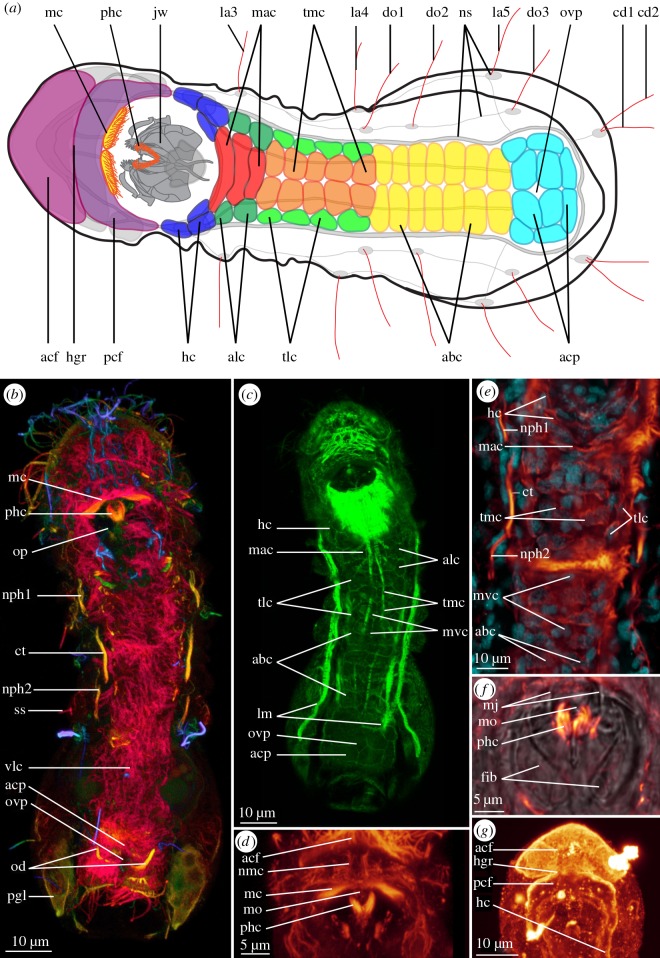
Ciliation of *Limnognathia maerski*. (*a*) Schematic drawing of the ventral locomotory ciliation of *L. maerski*. Ciliation in various colours, nervous system in grey. (*b*,*c*) CLSM maximum intensity projections. (*b*) Maximum depth intensity projection of the acetylated α-tubulin-LIR. (*c*) Ventral locomotory cell borders as seen with phalloidin in green. (*d*) Details of the head ciliation as seen with acetylated α-tubulin-LIR. (*e*) Details of the trunk ciliation as seen with acetylated α-tubulin-LIR in glow and DAPI in cyan. (*f*) Details of the relative position of the pharyngeal cilia as seen with acetylated α-tubulin-LIR in glow and transmitted light in grey. (*g*) Details of the head ciliated areas as seen with serotonin-LIR, in glow. Anterior end of specimen pointing left for (*a*), and to the top for (*b*–*g*). abc, abdominal ciliophores; acf, anterior ciliated field; acp, adhesive ciliary pad; alc, anterior lateral ciliophores ct, collecting tubule; cd1,2, caudalia 1 and 2; do1--3, dorsalia 1 to 3; fib, fibularium; hc, head ciliophores; hgr, head groove; jw, jaw; la3–5, lateralia 3 to 5; lm, longitudinal muscles; mac, median anterior ciliophores; mc, mouth ciliation; mj, main jaw; mo, mouth opening; mvc, medio-ventral aciliated cells; nmc, nerves of the mouth ciliation; nph1,2, protonephridia 1 and 2; ns, nervous system; od, oviduct; op, oral plate; ovp, ovipore; pcf, posterior ciliated field; pgl, posterior gland; phc, pharyngeal cilia; ss, sensorium; tlc, trunk lateral ciliophores; tmc, trunk median ciliophores; vlc, ventral locomotory ciliophores.

A pair of longitudinal ventro-median nerves (vmn, figures 1*a*–*c*,*f* and 2*a*,*h*) extends from the sub-pharyngeal ganglia. They are each about 1 µm wide, extend mid-ventrally along the thorax and the anterior part of the abdomen; laterally lining the ventral ciliation, and reaching the anterior edge of the adhesive ciliary pad. Co-localization with phalloidin staining shows that the ventro-median nerve is adjacent to the ventro-median muscle (mvm and vmn, figure 2*h*, and see [11]). We, therefore, suggest that the ventro-median nerves innervate the thoracic median ciliophores (tmc, figure 3*a*,*c*,*e*), the abdominal ciliophores (abc, figure 3*a*,*c*,*e*) and the ventro-median longitudinal muscle.

All the nerves described above show acetylated α-tubulin-LIR. Serotonin-LIR is found in the circumesophageal connective, the ventro-lateral nerve cords and the ventro-median longitudinal nerves, as well as in the perikarya of the brain and of the pharyngeal ganglion (described below). None of the longitudinal nerves show FMRF-amide-LIR.

#### 3.1.2. Peripheral nerves and sensoria

Along the lateral sides of the thorax and the abdomen, several pairs of cells show acetylated α-tubulin-LIR, each bearing one sensory cilium (= sensorium) (ss, figures 1*a*,*d*,*e*, 2*a* and 3*b*). We assume that as for Rotifera [24], each sensorium is a ciliated nerve cell, projecting axons towards the central nervous system; the axons and possibly interneurons constituting the peripheral nervous system (pns, figures 1*a*,*d* and 2*a*). Following the nomenclature of Kristensen & Funch [1], the sensoria are present as three pairs of lateralia (la3--5, figure 3*a*; la1--2 could not be found), three pairs of dorsalia (do1--3, figure 3*a*) and two pairs of caudalia (dorsal and ventral, cd1--2, figure 3*a*). Perikarya (scb, figure 1*a*,*d*) of five previously described additional sensoria could not be identified with acetylated α-tubulin-LIR. On each lateral side, the lateralia 3–5 as well as dorsalia 1–3 seem to project axons into one longitudinal dorso-lateral and one lateral neurite bundle, respectively, which meet up in the thorax and together join the circumesophageal connectives, anterior of the sub-pharyngeal ganglia. An additional branch of these peripheral nerves is found between lateralia 5 and the ventro-lateral nerve cord. Axons of the caudalia possibly connect to the posterior commissure; yet, this could not be ascertained due to the strong acetylated α-tubulin-LIR of the posterior glands.

#### 3.1.3. Brain

**Figure 4.**
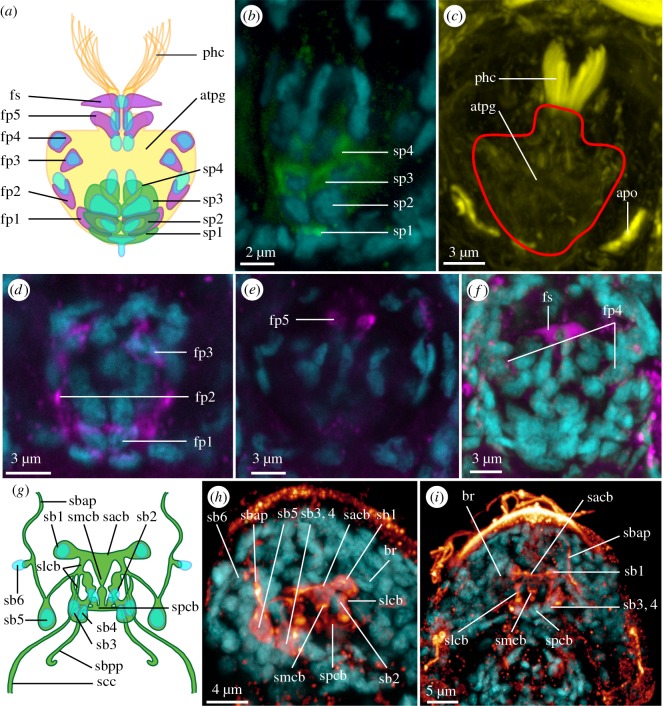
Details of the pharyngeal ganglion and the serotonin-LI-reactive brain of *Limnognathia maerski*. (*a*,*g*) Schematic drawings with acetylated α-tubulin-LIR in yellow, FMRF-amide-LIR in purple, DAPI in blue and serotonin-LIR in green. (*b*–*f* and *h*,*i*) CLSM maximum intensity projection with acetylated α-tubulin-LIR in yellow, FRMF-amide-LIR in purple, DAPI in cyan, and serotonin-LIR in green in (*b*) and in glow in (*h*) and (*i*). (*a*) Schematic drawing of the pharyngeal ganglion. (*b*) Details of the serotonin-LIR of the pharyngeal ganglion. (*c*) Overview of the acetylated α-tubulin-LIR of the pharyngeal ganglion. (*d*,*e*,*f*) Successive sub-stacks of the ventral, median and dorsal sections of the pharyngeal ganglion as seen with FMRF-amide-LIR. (*g*) Schematic drawing of the serotonin-LI-reactive brain. (*h*) Details of the serotonin-LI-reactive brain. (*i*) Overview of the serotonin-LI-reactive brain. Anterior end of specimens pointing to the top on all figures. apo, acetylated α-tubulin-LI-reactive pharyngeal organ; atpg, acetylated α-tubulin-LI-reactive pharyngeal ganglion; br, brain; fp1–5, FMRF-amide-LI-reactive perikarya of the pharyngeal ganglion; fs, FMRF-amide-LI-reactive spot of the pharyngeal ganglion; phc, pharyngeal cilia; s1–4, serotonin-LI-reactive perikarya of the pharyngeal ganglion; sacb, serotonin-LI-reactive anterior commissure of the brain; sb1–6, serotonin-LI-reactive perikarya of the brain; sbap, serotonin-LI-reactive brain antero-lateral nerve projection; sbpp, serotonin-LI-reactive brain posterior projection; scc, serotonin-LI-reactive circumesophageal connective; slcb, serotonin-LI-reactive lateral connective of the commissure of the brain; smcb, serotonin-LI-reactive median connective of the commissure of the brain; spcb, serotonin-LI-reactive posterior commissure of the brain.

The compact, undivided brain occupies most of the head (br, figures 1*a*,*e*, 2*a*,*b*,*g* and 4*h*,*i*). It was visualized with DAPI, acetylated α-tubulin-LIR, serotonin-LIR and FMRF-amide-LIR.

##### 3.1.3.1. DAPI

The brain (br, figures 1*a*,*e*, 2*a*,*b*,*g* and 4*h*,*i*) consists of densely packed small perikarya with small nuclei (nuclei diameter 1.5 to 2.5 µm, almost indistinguishable from each other) surrounding the neuropil. In the centre of the brain, slightly dorsally, is an area free of nuclei (measuring 6–7 µm longitudinally and 10–13 µm laterally) corresponding to the space occupied by the neuropil. Two auxiliary ganglia (ag, figures 1*a* and 2*a*,*g*) are present postero-lateral to the brain, each consisting of approximately 10 densely packed, small nuclei.

##### 3.1.3.2. Acetylated α-tubulin-LIR

Fine details of the acetylated α-tubulin-LIR were difficult to interpret due to the very diffuse IR; however, few structures could be described: A triangular neuropil is present centrally in the brain (np, figures 1*a* and 2*a*,*b*,*f*), which seems to comprise two very faint and diffuse anterior and posterior commissures. Each of them supplies a paired nerve extending ventro-posteriorly, the lateral-most nerve supplies the auxiliary ganglion of the brain (ag, figure 1*a*), after which they fuse into a circumesophageal connective (cc, figures 1*a*,*b*,*f* and 2*a*,*f*) lateral to the pharynx. Ventro-posterior of the brain, a pair of short nerves of the mouth ciliation (nmc, figures 1*a*, and 3*d*) innervates the paired ciliated tufts at the anterior edge of the mouth (mc, figures 1*a*, 2*a*,*b* and 3*a*,*b*,*d*, see below).

##### 3.1.3.3. Serotonin-LIR

Six pairs of serotonin-LI-reactive perikarya (sb1-6, figure 4*g*–*i*) are present around the serotonin-LI-reactive anterior and posterior commissures of the brain neuropil (sacb and spcb, figure 4*g*–*i*): one lateral pair (sb1, figure 4*g*–*i*) projects neurites into the anterior commissure, and a pair of paramedian perikarya (sb2, figure 4*g*,*h*) sends neurites into the posterior commissure. Both commissures are connected by an unpaired serotonin-LI-reactive median and a paired serotonin-LI-reactive lateral connective (slcb and smcb, figure 4*g*–*i*). Two pairs of serotonin-LI-reactive nerves extend from the posterior commissure: one short pair of serotonin-LI-reactive brain posterior projections ending blindly (sbpp, figure 4*g*) and one pair of serotonin-LI-reactive circumesophageal connectives (scc, figure 4*g*); the latter corresponding to the inner-branch of the acetylated α-tubulin-LI-reactive circumesophageal connective (cc, figures 1*a* and 2*a*,*f*). A cluster of two serotonin-LI-reactive perikarya (sb3--4, figure 4*g*–*i*) is present on each side, postero-lateral to the posterior commissure, which sends a pair of anterior projections to join the lateral connective of the brain. Finally, two pairs of perikarya (one large posterior (sb5, figure 4*g*,*h*) and one small anterior (sb6, figure 3*g*,*h*) supply a pair of serotonin-LI-reactive anterior projections (sbap, figure 3*g*–*i*) extending to the anterior margin of the animal.

##### 3.1.3.4. FMRF-amide-LIR

The brain shows a characteristic FMRF-amide-LIR pattern in the neuropil (figure 1*e*); however, due to the background signal of the anti-FMRF-amide staining, only one anterior pair of dorso-lateral FMRF-amide-LI-reactive brain perikarya could be identified (fbp, figure 1*e*), which is connected to the neuropil by an FMRF-amide-LI-reactive nerve.

#### 3.1.4. Sub-pharyngeal ganglia

A pair of presumed ventral sub-pharyngeal ganglia (spg, figures 1*a*,*f* and 2*a*) is found postero-laterally to the pharynx, supplying the circumesophageal connectives, the ventro-lateral nerve cords, the ventro-median nerves and the anterior commissure. They each contain approximately six to eight densely packed nuclei, which are distinguishable with DAPI staining, and found separate from adjacent neural and epidermal nuclei (no IR with the tested antibodies could be seen). Such ganglia were previously mentioned [23], though based on unpublished morphological observations, and more detailed transmission electron microscopy studies are still necessary to confirm the ganglionic arrangement of these DAPI-profiled perikarya.

#### 3.1.5. Pharyngeal ganglion

The pharyngeal ganglion is an unpaired cluster of nerve cells, surrounded by the fibularium sclerite, and situated dorso-posteriorly in the pharynx (pg, figures 1*a*–*c*,*e*, 2*a*,*b*,*g* and 4*a*–*f*), probably innervating the jaw elements. It shows positive IR for all antibodies tested (directed against acetylated α-tubulin, serotonin and FMRF-amide), revealing a consistent number and location of nuclei (stained with DAPI) in all examined specimens. A dense, filamentous acetylated α-tubulin-LI-reactive net of nerve fibres infiltrates the entire structure, and allows the delimitation of the ganglion (atpg, figures 1*c*, 2*f* and 4*a*,*c*), together with the densely packed nuclei. Of the approximately 60 cells identified with DAPI-staining, three paired serotonin-LI-reactive perikarya are clustered postero-medially in two longitudinal rows, followed by one unpaired serotonin-LI-reactive perikaryon (s1--4, figure 4*a*,*b*) and four pairs of FMRF-amide-LI-reactive perikarya which are found at the lateral and posterior margins of the pharyngeal ganglion (fp1--4, figure 4*a*,*d*,*f*) as well as one dorso-anterior pair of perikarya (fp5, figure 4*a*,*e*) and a pair of anterior FMRF-amide-LI-reactive positive spots lacking associated nuclei (fs, figure 4*a*,*f*).

How the pharyngeal ganglion is related to the central nervous system could not be resolved, as no nerves extending out of the pharyngeal ganglion could be identified. One pair of tufts of presumably pharyngeal sensory cilia (described below) originate directly from the pharyngeal ganglion (phc, figures 1*a*,*b*,*f*, 2*a*,*b*, 3*a*,*b*,*d*,*f* and 4*a*,*c*). One pair of strongly acetylated α-tubulin-LI-reactive structures are found postero-laterally to the pharyngeal ganglion, they do not seem to consist of cilia, and their function is unknown (apo, figures 1*b*, 2*f* and 4*c*).

### 3.2. Ciliation

The ciliation can be separated into five different systems: the external ventral locomotory ciliation, mouth ciliation, sensory cilia, the internally ciliated nephridia and oviducts.

#### 3.2.1. Locomotory ciliation

##### 3.2.1.1. Head ciliation

On the head, the ventral ciliation can be divided into a semicircular anterior ciliated field in front of the mouth opening (acf, figures 2*a*,*b* and 3*a*,*d*,*g*) separated by a transverse head groove (hgr, figures 2*a*,*b* and 3*a*,*g*) from a horseshoe-shaped posterior ciliated field (pcf, figures 2*a*,*b* and 3*a*,*g*) surrounding the mouth.

##### 3.2.1.2. Ciliophores

Acetylated α-tubulin-LIR, as well as phalloidin staining proved useful to reconstruct the ventral ciliary pattern of *L. maerski*. The packed cilia of each ciliophore could be differentiated in optical sections with acetylated α-tubulin-LIR, supported by phalloidin staining, which weakly marks the ventral cell walls. This showed that instead of one longitudinal row of paired ciliophores as previously described [1], the trunk ciliation consists of a more complex pattern at the anterior part of the thorax.

At the posterior part of the head and the anterior part of the thorax, the organization of the ciliophores is the most complicated. All four pairs of head ciliophores described in the original description of *L. maerski,* which were supposed to be lining the oral plate, could not be found. However, one pair of head ciliophores (hc, figure 3*a*,*c*,*e*,*g*) could be found, followed by two pairs of laterally adjacent ciliophores. These three pairs of ciliophores are likely to correspond to some of the head ciliophores described by Kristensen & Funch [1]. Three unpaired, transversely elongated ciliophores (mac, figure 3*a*,*c*,*e*) and two pairs of antero-lateral ciliophores (alc, figure 3*a*,*c*) are found posterior to the oral plate. More posteriorly, on the thorax, two paired longitudinal rows of ciliophores are present: one row of four lateral ciliophores (tlc, figure 3*a*,*c*,*e*) and one row of five median ciliophores (tmc, figure 3*a*,*c*,*e*). The row of thoracic lateral ciliophores is in tight contact with the thoracic median row of ciliophores, giving a mosaic appearance, probably explaining the previous indiscernibility of each row. The cells of the median row are larger, and are adjacent to the midline. The thoracic lateral ciliophores are smaller, and each of them is in contact with two thoracic median ciliophores. At the posterior part of the thorax and the anterior part of the abdomen, only two longitudinal rows exist, each consisting of six abdominal ciliophores (abc, figure 3*a*,*c*,*e*), corresponding to the observations of the original description [1]. On the midline between each median quartet of ciliophores, one small non-ciliated epidermal ventro-median cell (mvc, figure 3*c*,*d*) is present.

##### 3.2.1.3. Adhesive ciliary pad

The ciliary adhesive pad (acp, figures 1*a*,*g*, 2*a*,*b* and 3*a*–*c*) consists of five pairs of multi-ciliated cells: two lateral, two median and one posterior, as described in the original description [1].

#### 3.2.2. Mouth ciliation

In accordance with the original description [1], a mouth ciliation is found most likely involved in food uptake. However, it covers only the anterior edge of the mouth cavity, comprising paired laterally elongated tufts of more than 10 approximately 7 µm long cilia (mc, figures 1*a*, 2*a*,*b* and 3*a*,*b*,*d*).

This CLSM study revealed a conspicuous previously undescribed pharyngeal ciliary tuft in the mouth cavity (phc, figures 1*a*,*b*,*f*, 2*a*,*b*, 3*a*,*b*,*d*,*f* and 4*a*,*c*). It extends between the main jaws (mj, figure 3*f*), and its ciliary roots originate from the pharyngeal ganglion, suggesting that the cilia have sensory function. The cilia are 6–7 µm long and curved (phc, figures 2*b*, 3*a*,*b*,*d*,*f* and 4*c*), and each of the paired tufts consists of more than 10 cilia, as also seen in the TEM micrographs shown in Kristensen & Funch ([1], fig. 23) and Sørensen & Kristensen ([23], fig. 3.12.B)

#### 3.2.3. Nephridia

Three pairs of acetylated α-tubulin-LI-reactive ventro-lateral longitudinal ciliary structures are found along the thorax and anterior abdomen. The present CLSM data in combination with the TEM data of Sørensen and Kristensen [23] allow us to reconstruct these structures as an anterior and a posterior pair of protonephridia with an intermediate collecting tubule, in accordance with Sørensen and Kristensen [23] but opposing the interpretation of three pairs of protonephridia given by Sørensen *et al*. [25] (figs. 16.13 and 16.15). This study offers the following more detailed description.

The anterior pair of protonephridia originates in the anterior-most thorax, each protonephridium comprising two adjacent protonephridial units (pu1--2, figure 2*i*,*j*) with two monociliated terminal cells each; all four cilia (8–10 µm long) joining in one common canal cell (nph1, figures 1*b*, 2*a*,*i*,*j* 3*b*,*e* and fig. 3.15 in Sørensen and Kristensen [23]). The posterior pair of protonephridia (nph2, figures 1*b*, 2*a*,*i*,*j* and 3*b*,*e*) contains only one unit (pu2, figure 2*i*,*j*, contrary to the double units proposed by Sørensen & Kristensen [23], but see fig. 3.15B) with two monociliated terminal cells (cilia 7–10 µm long), possibly originating in the anterior abdomen and extending anteriorly into the posterior thorax, where it meets the collecting tubule. The intermediate collecting tubule (ct, figures 1*b*, 2*a*,*i*,*j* and 3*b*,*e*) comprises more than five tightly packed cilia, but the exact number could not be determined. It extends through the second third of the thorax and is 11–13 µm long. The consistent longer length of the cilia of the collecting tubule and higher cilia density, similar to what is shown in Sørensen & Kristensen [23], are elements allowing us to differentiate these collecting tubules from the terminal cells. No associated nephridiopore or additional structure could be found.

#### 3.2.4. Oviducts

One pair of acetylated α-tubulin-LI-reactive L-shaped ducts here interpreted as oviducts (od, figures 2*a* and 3*b*) is present in the posterior part of the abdomen, but does not consist of cilia. They originate lateral to the midline, posterior to the oocyte, extend 6–7 µm postero-medially, terminating in an ovipore (ovp, figure 3*a*,*b*,*c*) in the centre of the adhesive ciliary pad. Non-ciliated oviducts are also reported in Rotifera [26–28], whereas nothing similar has been found in Gnathostomulida [29,30].

Anterior of the oviduct, a pair of putatively associated 10 µm long dorsal accessory cilia (aco, figure 2*a*,*e*) is present. Each cilium is adjacent to the oocyte; oriented obliquely, extending from a dorso-median to a ventro-lateral position. Their function is unknown.

### 3.3. Glands

A tripartite glandular complex consisting of a central gland (mhg, figure 2*a*,*c*,*d*) and a pair of lateral, elongated glands (lhg, figure 2*a*,*c*,*d*) are found in the dorsal head region of *L. maerski* (hg, figure 2*b*). All glands show acetylated α-tubulin-LIR in the cell wall, and appear to open dorso-apically on the head. The median gland extends dorso-posteriorly to the level of the pharynx, and possesses numerous and densely packed nuclei (mhg, figure 2*c*). The two lateral glands consist of an elongated longitudinal canal anteriorly embedding few elongated nuclei (lhg, figure 2*c*), which extends posterior of the median gland until the dorso-lateral sides of the pharynx (figure 2*d*).

One pair of large glandular cells is found ventro-laterally in the posterior-most abdomen (pgl, figures 1*a*, 2*a*,*e* and 3*b*); their full configuration was detected through background signal of non-specific fluorescence as well as specific acetylated α-tubulin-LIR. Each cell is 15 to 20 µm long, ellipsoid shaped, broadest at its base and narrowing into a neck region, with a 2 µm wide opening; the elongated nucleus is positioned at the external side of the cell (npg, figure 2*e*). The cell wall of the neck region contains numerous, distinct acetylated α-tubulin-LIR longitudinal components; their signal becoming less obvious towards the expanded cellular base. FMRF-amide-LIR and serotonin-LIR are visible in the cell opening (opg, figure 2*a*,*e*). Their position corresponds to the ‘paired openings of unknown function’ of Sørensen & Kristensen [23] visible with SEM, which therefore are not nephridiopores as previously suggested.

## 4. Discussion

### 4.1. Evolution of ventral cords and associated commissures in Gnathifera

The presence of two ventro-lateral nerve cords in *L. maerski* was confirmed [1,23], and their precise configuration was explained, unravelling an anterior (possibly with associated sub-pharyngeal ganglia) and a posterior commissure, as well as two ventro-median nerves branching off from the main ventro-lateral cords at the sub-pharyngeal ganglia; these paired ventro-median nerves are not previously reported in Gnathifera.

**Figure 5.**
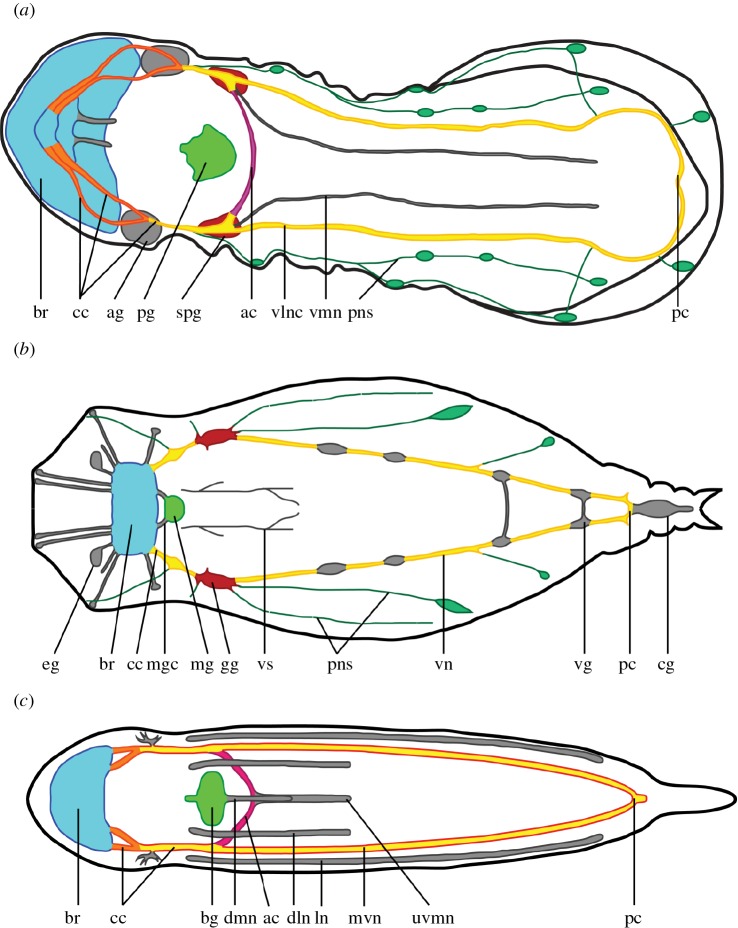
Comparison of the nervous system of Gnathifera. Schematic drawing of the dorsal view of the nervous system of three Gnathifera. Different colours represent parts of the nervous system that may be homologous between the different animals, but see the text for a full discussion. Grey structures are parts of the nervous system that cannot be homologized. Anterior end pointing left on all figures. (*a*) Micrognathozoa: *Limnognathia maerski*. (*b*) Rotifera, Monogononta, modified from Remane 1933 [34]. (*c*) Gnathostomulida: *Gnathostomula peregrina*, modified from Müller and Sterrer, 2004 [10]. ac, anterior commissure; ag, auxiliary ganglion; br, brain; bg, buccal ganglion; cc, circumesophageal connective; cg, caudal ganglion; dln, dorso-lateral nerve; dmn, dorso-median nerve; eg, epipharyngeal ganglion; gg, geniculate ganglion; ln, lateral nerve; mg, mastax ganglion; mgc, mastax ganglion connective; mvn, main ventral nerve; pc, posterior commissure; pg, pharyngeal ganglion, pns, peripheral nervous system; spg, sub-pharyngeal ganglion; uvmn, unpaired ventro-median nerve; vg, vesicular ganglion; vlnc, ventro-lateral nerve cord; vmn, ventro-median nerve; vn, ventral nerve; vs, visceral nerve.

In Rotifera, only one pair of longitudinal ventro-lateral nerves has been consistently found with FMRF-amide-LIR, catecholamine-LIR, serotonin-LIR and SCPb-LIR in representatives of both Bdelloidea and Monogononta [12–15,26,31,32]. TEM investigations by Ahlrichs ([33]; figure 5*a*) also suggest the presence of at least two longitudinal nerves in the neck region of the early branching Seisonidea. However, antibody-staining shows only a subset of the nervous system, and acetylated α-tubulin-LIR has not been tested in these studies. Yet, in a total reconstruction of the nervous system of Monogononta based on light microscopy by Remane [34] (figure 5*b*), no ventro-median nerves were found even though more delicate nerves were described, such as the peripheral nerves. These have been shown to branch off dorso-laterally from the sub-pharyngeal ganglia and innervate the sensory organs and dorso-ventral muscles [35], similar to what is here described for *L. maerski*. Although no anterior commissure and ganglia resembling those of *L. maerski* are generally found in Rotifera, similarities can exceptionally be found in the FMRF-amide-LI-reactive and SCPb-LI-reactive perikarya and trunk commissure in *Notommata copeus* Ehrenberg, 1834 [36] (Monogononta) [13], the FMRF-amide-LI-reactive trunk commissure in *Euchlanis dilatata* (Ehrenberg, 1832) [14,37], or the so-called geniculate ganglion of Monogononta [34] (figure 5*b*).

In Gnathostomulida, confocal and TEM studies show a more variable number of one to three pairs of longitudinal nerves (plus one dorsal and one median unpaired nerve in *Gnathostomula peregrina* Kirsteuer, 1969 [38] (figure 5*c*)), of which the paired ventro-lateral nerves form an anterior as well as a posterior commissure in *G. peregrina* [10]. Similar to *L. maerski*, their circumesophageal connectives also originate as two distinct bundles of neurites in *G. peregrina* [10] (figure 5*a*,*c*), further supporting the homology of two ventro-lateral cords in Gnathifera. This character is likely to be shared between most Spiralia [39], suggesting that the ventro-lateral nerve cord of Gnathifera is possibly a symplesiomorphy of this group. However, with Gnathostomulida being sister group to the remaining Gnathifera [3] and the only sporadic finding of an anterior commissure and/or sub-pharyngeal ganglia in both Gnathostomulida and Rotifera, the homology of these specific substructures of the ventral cords remains to be tested. The reported ventro-median nerve in *G. peregrina* is unpaired, but two separate ventro-median strands originating in the anterior trunk are observed in new ongoing studies of other gnathostomulids (Gąsiorowski, N.B. and K.W. 2016, unpublished data), warranting further analyses of their possible homology to the ventro-median nerves of *L. maerski*.

#### 4.1.1. Finding of a synapomorphic pharyngeal ganglion with ciliary receptors in Gnathifera

This study confirms the presence of a formerly suspected pharyngeal ganglion in *L. maerski* [23] with numerous nucleated cells, an observation refuting the suggestion of Gorelick [40], proposing that ‘Micrognathozoan jaws may also be enervated by anucleate neurons’. In Rotifera, a ‘mastax ganglion’ is suspected but not yet confirmed in Seisonidae [26], and data are scarce on Bdelloidea because only the presence of catecholaminergic nerves related to the mastax suggests its presence in *Rotatoria tardigrada* Ehrenberg, 1832 [31,37]. However, for Monogononta, this ganglion has shown IR for serotonin, catecholamines and FMRF-amide [13,14,31,41]. Yet, IR, nerves and perikarya repartition are extremely variable, and no detailed comparison with *L. maerski* is possible. In Gnathostomulida, Herlyn & Ehlers [42] reject the presence of a buccal ganglion after failing to find any correspondent structures in *Gnathostomula paradoxa*. However, other researches do not support this conclusion, and the so-called buccal ganglion has been described in Filospermoidea with TEM [20] and in Bursovaginoidea with TEM and CLSM [10,16,18,20]. CLSM studies [10,16] further show the presence of FMRF-amide-LI-reactive perikarya in the buccal ganglion of *Gnathostomula peregrina*. Although connections between the central nervous system and the pharyngeal ganglion of *L. maerski* have not been found, studies of Gnathostomulida [16,20] and Rotifera [31] indicate that a pair of nerves originates dorso-laterally from the posterior of the brain, supplying the buccal/mastax ganglion (mgc, figure 5*b*). This study, as well as the literature, indicates that different homologues of the pharyngeal ganglion of *L. maerski* are found in most Gnathifera (figure 5), thus this character might be a synapomorphy of this group.

The here discovered pharyngeal cilia extending between the main jaws in *L. maerski* can actually be recovered in previously published transmission electron micrographs, such as figs. 23 and 25 in [1]. Intriguingly, sensory cilia with similar position, innervation and configuration are also found in rotifers, such as the Bdelloidea (*Philodina roseola* Ehrenberg, 1832 [24,37] and *Philodina acuticornis odiosa* Milne, 1916 [43,44]), or Monogononta (*Asplanchna brightwellii* Gosse, 1850 [24,45]). These cilia likewise protrude between the basal parts of the rami (assumedly homologous to the main jaws of *L. maerski*), and are also anchored at the mastax ganglion (assumedly homologous to the pharyngeal ganglion in *L. maerski*). Additionally, in *Asplanchna brightwellii* the distal part of the ciliated sensory receptors is well separated into two bundles, resembling the paired configuration in *L. maerski* [24]; all supporting their homology and their organization into densely ciliated tufts as a putative synapomorphy of Micrognathozoa and Rotifera. Although so far, data on the early branching rotifer Seisonidae are lacking. In Gnathostomulida, pharyngeal ciliation has never been described; however, scarce cilia are visible in the pharynx of *Gnathostomula paradoxa* ([42]: fig. 3), and ongoing investigations indicate the existence of possible homologous short paired ciliary receptors, between the jaws connected to the so-called buccal ganglion in *Gnathostomula paradoxa*, *Austrognathia microconulifera* Farris, 1977 [46] and *Haplognathia* spp*.* (Gąsiorowski, N.B. and K.W. 2016, unpublished data). The putative common presence of paired ciliary receptors on the pharyngeal ganglia across Gnathifera, thereby further supports the homology of the pharyngeal ganglion (as well as its possibly common sensory function) in Gnathifera.

### 4.2. Increased resolution of ciliary patterns revealed with high-quality confocal laser scanning microscopy

Acetylated α-tubulin-LIR as well as phalloidin and DAPI show a more complex pattern of ventral ciliophores than previously described in *L. maerski* [1]. These results show the relevance of CLSM to resolve spatial patterns in microscopic animals as the collapse of cilia makes difficult the identification of independent cells with light microscopy or scanning electron microscopy. Similar complex anterior ciliary arrangements have been found in the gastrotrichs *Diuronotus aspetos* Todaro, Balsamo & Kristensen, 2005 [47,48], *Diplodasys rothei* Kieneke, Narkus, Hochberg & Schmidt-Rhaesa, 2013 [49] or the microscopic annelids *Diurodrilus* spp. [50]. Interestingly, *L. maerski* and *Diurodrilus* have been comprehensively compared [1,50], and even though phylogenomics recently confirmed that *Diurodrilus* is a distantly related genus of annelids [2,51], this is another similar character between these two animals. However, although the overall similarity in patterns may reflect homoplasy, the detailed patterns have been shown to be of systematic significance within, e.g. *Diurodrilus* and Gastrotricha [50,52,53], and may also potentially be useful for discriminating Micrognathozoa from Greenland versus Antarctica, which was not possible according to jaw morphology [21].

### 4.3. Protonephridial system of Micrognathozoa shows more similarity with Rotifera than Gnathostomulida

The protonephridial system of *L. maerski* resembles the one of Rotifera, although only few studies have reconstructed the excretory system of Rotifera in detail. However, Ahlrichs provided the complete reconstruction of the protonephridial system of *Paraseison annulatus* (Claus, 1876) [54] (Seisonidae) [33] and *Proales reinhardtii* (Ehrenberg, 1834) [36] (Monogononta) [55] from ultrathin section and TEM. Both rotifers possess a terminal syncytium with several multi-ciliated terminal organs and a capillary canal (resembling the canal cell in *L. maerski*). Furthermore, the terminal syncytium connects to a multi-ciliated canal region, which shows resemblance to the collecting tubules of *L. maerski.* The main difference in this configuration is the monociliated nature of the terminal organs of *L. maerski* versus the multi-ciliated organs found in most rotifers [33,35,55,56]. The protonephridial system of Gnathostomulida has been described in detail for *Haplognathia rosea* (Sterrer, 1969) [19] (Filospermoidea) and *Gnathostomula paradoxa* by Lammert [20]. They consist of serially independent organs, each comprising a monociliated terminal cell, a canal cell and a nephridiopore cell; an arrangement found in other animals [57,58]. Therefore, it can be assumed that the monociliated terminal cells of *L. maerski* are a plesiomorphic condition shared with Gnathostomulida, whereas the multi-ciliated collecting tubule supplying the different canal cells may be a synapomorphy of *L. maerski* and Rotifera.

### 4.4. Do Micrognathozoa possess a retrocerebral organ?

The tripartite anterior gland of *L. maerski*, consisting of one unpaired median and a pair of medio-lateral glands opening dorso-apically is very similar in position and size to the retrocerebral organ found in most Rotifera, where they are assumed to play a role in the lubrication of the cilia [24,26,35]. If the two organs are homologous, the median gland of *L. maerski* would correspond to the retrocerebral sac, while the lateral glands would correspond to the sub-cerebral glands more similar to what is found in Bdelloidea [35] (where the retrocerebral sac likewise opens medially and the two sub-cerebral glands open medio-laterally), hereby indicating that this may be the plesiomorphic condition in Rotifera, and that the retrocerebral organ might be a synapomorphy of Micrognathozoa and Rotifera.

## 5. Conclusion

This study shows a striking simplicity of the micrognathozoan nervous system, in opposition to the complexity in muscular [11] and ciliary systems (this study), but it also illustrates the need for CLSM studies together with TEM investigations on meiofaunal animals. Indeed, previous TEM studies on Micrognathozoa did not uncover the second ventro-median pair of longitudinal nerves or the exact details of the ventral ciliation. On the other hand, some conclusions of this paper could not have been possible without previous TEM studies, such as the identification of the protonephridial unit versus the collecting tubule.

Indeed, many characters described in this study seem to be autapomorphies of Micrognathozoa, such as the presence of a paired ventro-median nerve, or the specific arrangement of ciliophores. On the other hand, some characters constitute putative synapomorphies of Micrognathozoa and Rotifera, such as the peripheral nervous system innervating the sensory structures, the presence of dense tufts of pharyngeal sensory cilia, the organization of the protonephridia and the potential presence of a retrocerebral organ. Furthermore, resolving the morphology of the nervous system of Micrognathozoa allowed us to hypothesize that a ciliated pharyngeal ganglion is a synapomorphy of all Gnathifera, and that the presence of two ventro-lateral nerve cords is a symplesiomorphy of Gnathifera, and more generally of Spiralia [39].

Although this study informs on the inner anatomy of Micrognathozoa, many details still warrant further ultrastructural studies, such as the protonephridia and the oviducts, or the connection of the pharyngeal ganglion to the brain. Additionally, detailed CLSM studies are lacking on many Gnathifera, such as the rotifer group Seisonidae, where only the musculature has been described [7], and the gnathostomulid groups Filospermoidea and Conophoralia. In the context of the latest phylogenomic results [2,3] where Gnathifera has a key phylogenetic position within protostomes, we hope that these issues will soon be addressed.

## Data accessibility

Confocal stacks readable with the Imaris software (Bitplane Scientific Software, Zürich, Switzerland) [59] are accessible on Dryad Digital Repository at http://dx.doi.org/10.5061/dryad.n3vn8.
